# Genome-Wide Identification of Binding Sites for SmTCP7a Transcription Factors of Eggplant during Bacterial Wilt Resistance by ChIP-Seq

**DOI:** 10.3390/ijms23126844

**Published:** 2022-06-20

**Authors:** Xi’ou Xiao, Wenqiu Lin, Enyou Feng, Caiyu Wu, Xiongchang Ou

**Affiliations:** 1 South Subtropical Crop Research Institute Chinese, Academy of Tropical Agricultural Sciences, Zhanjiang 524091, China; linwenqiu1989@163.com (W.L.); wucaiyu1995@163.com (C.W.); oxc9527@163.com (X.O.); 2 Key Laboratory for Post-harvest Physiology and Technology of Tropical Horticultural Products of Hainan, Zhanjiang 524091, China; 3 Zhanjiang City Key Laboratory for Tropical Crops Genetic Improvement, Zhanjiang 524091, China; 4 Zhanjiang Academy of Agricultural Sciences, Zhanjiang 524091, China; enyou2008@163.com

**Keywords:** gene regulation, MAP kinase, *R. solanacearum*, resistance, TCP transcription factors

## Abstract

Teosinte branched 1/cycloidea/proliferating cell factor (TCP) transcription factors play a key role in the regulation of plant biotic and abiotic stresses. In this study, our results show that SmTCP7a positively regulated bacterial wilt that was caused by *Ralstonia solanacearum.* ChIP-seq was conducted to analyze the transcriptional regulation mechanism of SmTCP7a before (R0 h) and 48 h after infection (R48 h). SmTCP7a regulated a total of 92 and 91 peak-associated genes in R0 h and R48 h, respectively. A KEGG (Kyoto encyclopedia of genes and genomes) pathway analysis showed that phenylpropanoid biosynthesis, MAPK (mitogen-activated protein kinas) signaling pathway, plant hormone signal transduction and plant-pathogen interactions were involved. The difference in peaks between R0 h and R48 h showed that there were three peak-associated genes that were modulated by infection. A better understanding of the potential target genes of SmTCP7a in response to *R. solanacearum* will provide a comprehensive understanding of the SmTCP7a regulatory mechanism during the eggplant defense response to bacterial wilt.

## 1. Introduction

TCP transcription factor, which contains a basic helix–loop–helix DNA binding domain, the TCP domain, is a plant-specific transcription factor. Based on TCP domain differences, TCP families can be further divided into class I and class II. The conserved DNA binding site of class I and class II TCPs is GGNCCCAC and GTGGNCCC, respectively [1]. The TCPs play a key role in the regulation of plant growth and development, such as pistil abortion [2], leaf development [3], root growth [4], plant architecture [5,6] and so on. In *Arabidopsis*, AtTCPs can interact with 236 proteins involved in the biosynthesis of phytohormones and their signal transduction [7]. Several results have shown that the TCPs can directly regulate the biosynthesis and signaling pathways of SA(Salicylic acid), JA(Jasmonic acid), CK(Cytokinin), ABA(Abscisic acid) and BR(Brassinoids) [1]. In addition, TCPs can directly bind to promoters and regulate target gene expression, thereby positively or negatively regulating the biosynthesis of phytohormones [1,8]. In Arabidopsis, AtTCP4, AtTCP9 and AtTCP20 can bind to the *LOX2* promoter to regulate JA biosynthesis [9]. AtTCP8 can bind to the *ICS1* promoter to regulate SA biosynthesis [9,10] as well as the key BR transcriptional regulators, *BZR1* and *BZR2/BES1* [11]. However, the molecular mechanism underlying their transcription control remains unclear.

The TCPs not only regulate plant development, they can regulate plant biotic and abiotic stress responses [8,9,12,13,14,15,16]. In addition, TCPs can be recognized by pathogen effectors and can initiate plant defense responses [17]. Previous studies have shown that TCP13, TCP14, TCP15, TCP19 and TCP21 can interact with several pathogen effectors [18,19]. The *Phytophthora capsica* effector CRN12_997 can bindSlTCP14-2 and repress its transcriptional activity to reduce plant resistance [19]. TCPs can interact with other proteins and form a protein-complex, such as AtTCP8, AtTCP14 and AtTCP15 interacting with SRFR1 to regulate the defense response [14]. In addition, AtTCP8, AtTCP14 and AtTCP15 can physically interact with NPR1 to enhance the binding of the *PR5* promoter, which leads to systemic acquired resistance [13]. In cucumber, CsTCP14 can repress the expression of *CsNBS-LRR*, resulting in susceptibility to foliage diseases [20].

Eggplant (*Solanum melongena* L.) is an important vegetable in subtropical and tropical regions. It contains various phenolic compounds that can improve human health. However, bacterial wilt caused by *R.* *solanacearum* has caused severe yield reductions worldwide. Thus, several studies have been conducted to comprehensively understand the molecular mechanism of eggplant defense against *R. solanacearum*. Thousands of DEGs (differentially expressed genes) were identified by RNA-seq after eggplant inoculation with *R.* *solanacearum*. KEGG analysis of these DESs showed that the signaling pathways of phytohormones such as SA and JA play a vital role in the defense response [21]. Several transcriptional factors, such as MYB, NAC and WRKY, also regulate the defense response. The MYB transcriptional factor SmMYB44 directly regulates *SmSPDS* expression to increase spermidine content, which increases resistance to bacterial wilt [22]. By contrast, the NAC transcriptional factor SmNAC negatively regulates the expression of *ICS1* (isochorismate synthase 1) and reduces resistance to bacterial wilt [23]. Genome-wide analysis showed that *SiWRKY53* is a source of resistance to bacterial wilt [24]. Our previous study showed that reactive oxygen species are also involved in bacterial wilt resistance in eggplant [25]. Moreover, SmEDS1 (enhanced disease susceptibility 1), which is involved in the SA biosynthetic pathway, positively regulates bacterial wilt resistance [26]. A yeast two-hybrid study showed that SmEDS1 interacts with SmTCP7a [27]. In the present study, our results show that SmTCP7a positively regulates bacterial wilt resistance. Thus, ChIP-seq of SmTCP7a was analyzed to detect the specific genes regulated by SmTCP7a during the eggplant response to *R. solanacearum*. Our results identify several potential candidate genes, and provide a comprehensive understanding of genes regulated by SmTCP7a during eggplant defense against *R.* *solanacearum.*

## 2. Results

### 2.1. Molecular Characterization of SmTCP7a

**Figure 1:**
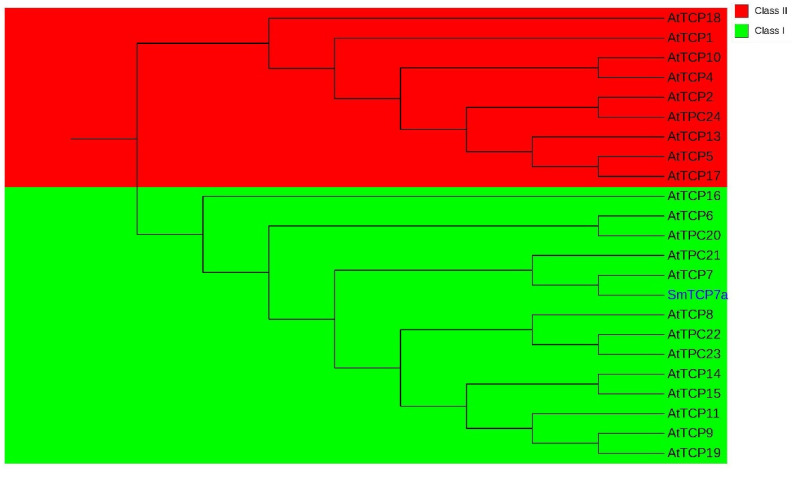
The phylogenetic tree for SmTCP7a. The phylogenetic tree was analyzed by NJ tree with 1000 bootstrap replicates.

**Figure 2:**
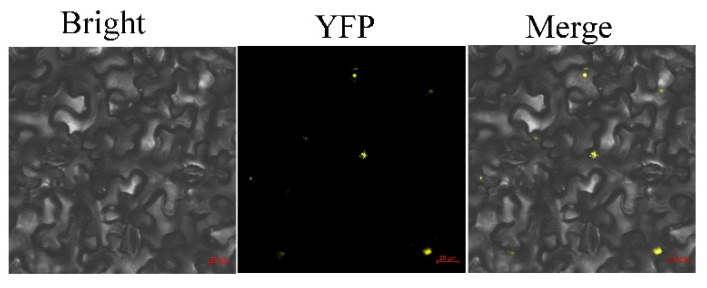
The subcellular location of SmTCP7a. SmTCP7a-YFP was detected using *Nicotiana benthamiana* leaves. Bar = 20 μm.

The CDS of SmTCP7a contains 783 bp, and codes 260 amino acids. The molecular weight of SmTCP7a was 27.09 kDa and the theoretical pI was 8.79. Phylogenetic analysis indicated that SmTCP7a belongs to class I TCPs (Figure 1). In addition, a subcellular localization assay showed that SmTCP7a is localized in the nucleus (Figure 2).

### 2.2. Analysis of SmTCP7a Transactivation Functions

**Figure 3:**
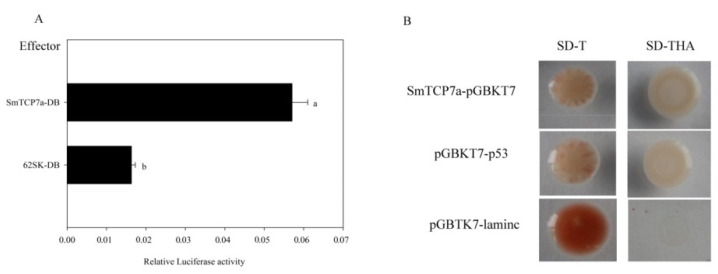
Analysis of SmTCP7a transactivation functions. (**A**) Transactivation of GAL4 response transcription by SmTCP7a fused to the DNA-binding domain of GAL4 in *N. benthamiana* leaves. Different letters indicate statistically significant difference (independent-sample *t*-test (confidence interval = 95%), *n* = 3, α = 0.05). The data were analyzed by the SPSS17 software. (**B**) Transactivation by SmTCP7a in a yeast one-hybrid.

Compared with 62SK-DB expression, SmTCP7a-DB stimulated the GAL4-response 2.49 times (Figure 3A), demonstrating that SmTCP7a is an activator of transcription. In yeast one-hybrid experiments, SmTCP7a-pGBKT7 and pGBKT7-p53 could grow in SD-T and SD-THA medium, whereas pGBKT7-laminC could grow in SD-T medium but not SD-THA medium (Figure 3B). This result also demonstrates that SmTCP7a is an activator of transcription.

### 2.3. SmTCP7a Positively Regulates Eggplant Bacterial Wilt Resistance

**Figure 4:**
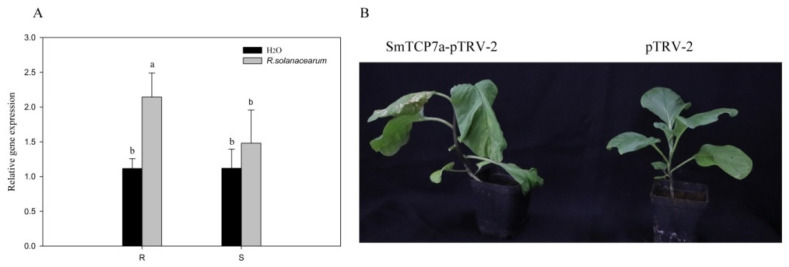
SmTCP7a positively regulates eggplant bacterial wilt resistance. (**A**) Analysis of the expression level of the SmTCP7a after inoculation with *R. solanacearum* at 48 hpi. Different letters indicate statistically significant difference (ANOVA, Duncan post hoc test, *n* = 3, α = 0.05). The data were analyzed by the SPSS17 software. (**B**) Eggplant wilted after VIGS of SmTCP7a. The photos were taken 7 days after inoculation with *R. solanacearum*.

After inoculation of eggplants with *R. solanacearum*, the SmTCP7a expression level significantly increased in the R genotype, but not in the S genotype (Figure 4A). The result of further VIGS (virus induced gene silencing) showed that SmTCP7a positively regulated bacterial wilt resistance in eggplant (Figure 4B).

### 2.4. ChIP-Seq Libraries and Detection of Peaks for SmTCP7a

Western-blot analysis with a polyclonal antibody showed the presence of the SmTCP7a target band (Figure S2). Eight ChIP-seq libraries were created for eggplants before (R0 h) and 48 h after infection (R48 h) with *R. solanacearum.* We obtained 82.0 million clean reads for the R0 h antibody-treated library compared to 82.6 million clean reads for the R0 h control sample (pre-immune sera). A total of 94.3 million clean reads was obtained for the R48 h antibody-treated library and 87.4 million clean reads for the R48 h control sample (Table 1).

**Figure 5:**
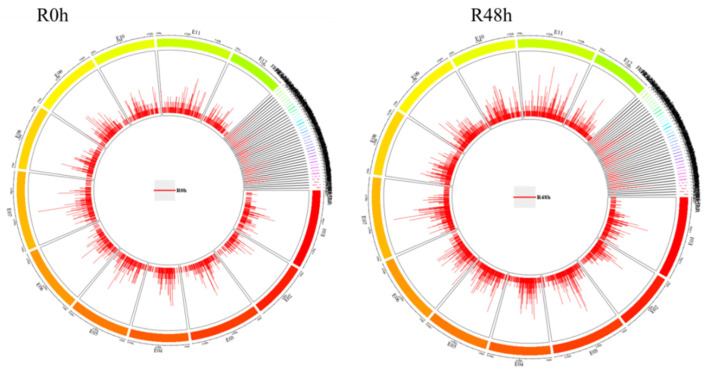
An overview of ChIP-Seq peak distribution in eggplant chromosomes for R0 h and R48 h.

After alignment to the eggplant reference genome, the peaks were called by MACS software with a *p*-value < 0.5 [28]. There was a total of 4378 peaks in the R0 h sample and 6555 peaks in the R48 h sample (Tables S1 and S2). The number of peaks in R48 h was higher than R0 h, and the distribution of the peaks in the genome is shown in Figure 5. In R0 h, the E7 and E11 chromosomes contained the most peaks (354) and the E2 chromosome contained the least (180 peaks). In R48 h, the E7 chromosome contained the most peaks (568) and the E2 chromosome contained the least (297 peaks).

### 2.5. Annotation of Detected Peaks and Discovery of Motifs in Promoter Regions

**Figure 6:**
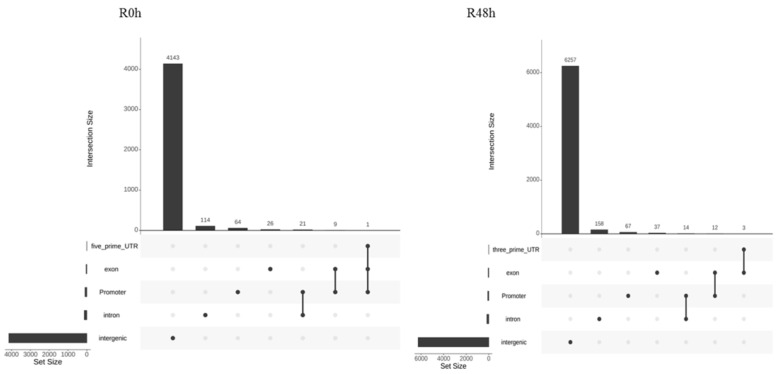
An overview of the genomic distribution of SmTCP7a binding sites for R0 h and R48 h.

**Figure 7:**
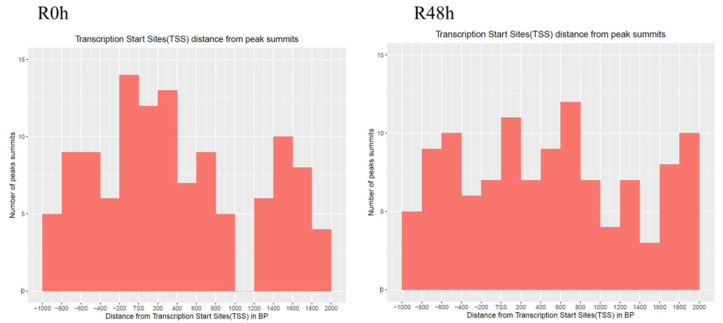
The distance between transcription start sites and MACS peak summits for the gene-associated peaks in R0 h and R48 h.

The genomic location of peaks was analyzed by using the ChIPseeker R package [29]. The result showed that about 94% of peaks were located in the intergenic region (Figure 6). Figure 7 shows that the peaks were located in close proximity to transcription start sites (TSS) in both R0 h and R48 h. In R0 h, 95 peaks were located in promoter regions and 92 genes were identified (Table S3). In R48 h, 93 peaks were located in promoter regions and 91 genes were identified (Table S4). Most of peak-associated genes were annotated as hypothetical proteins or uncharacterized proteins. Genes that may be related to plant disease resistance are listed in Table 2. Interestingly, histone H2A (Smechr0702191) was annotated in both R0 h and R48 h, whereas TCP9-like transcription factor was only detected in R48 h.

**Figure 8:**
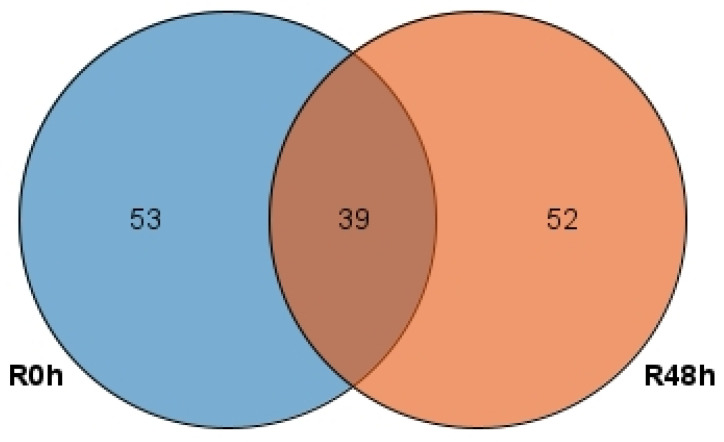
Venn diagram showing the overlap of peak-associated genes for R0 h and R48 h.

**Figure 9:**
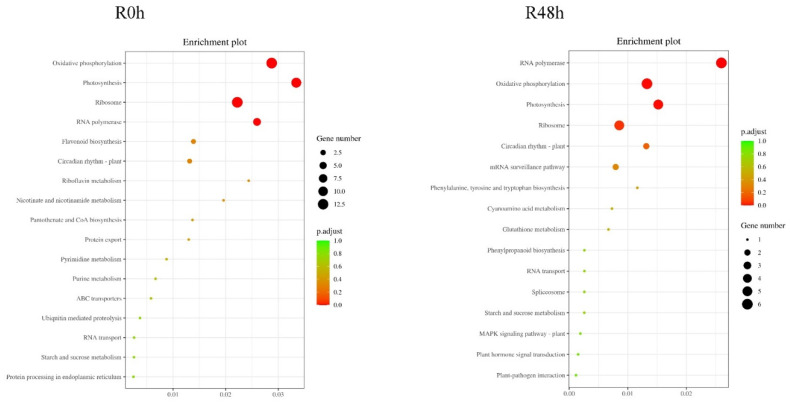
KEGG enrichment pathways for the peak-associated genes in R0 h and R48 h.

Figure 8 shows that 39 peak-associated genes were common to R0 h and R48 h. In addition, 53 genes and 52 genes were unique to R0 h and R48 h, respectively. The KEGG enrichment results showed that pathways for phenylpropanoid biosynthesis, MAPK signaling, plant hormone signal transduction and plant-pathogen interactions were active in R48 h (Figure 9). However, in R0 h only flavonoid biosynthesis may have been related to the defense response.

The motifs in the promoter region were analyzed using Homer software by de novo methods. There was a total of 17 and 22 predicted motifs in R0 h in R48 h, respectively, and only one motif (Bach2 (bZIP)/OCILy7-Bach2-ChIP-Seq (GSE44420)/Homer (0.746)) was identified in both R0 h and R48 h. All motifs were identified in JASPAR CORE plants or yeast, where two R48 h motifs were identified as TCP binding motifs (Tables S5 and S6).

### 2.6. Differences in Peaks between R0 h and R48 h

The difference in the peaks between R0 h and R48 h was detected by the Diffbind R package with a *p*-value < 0.05 and fold > 0 [30]. A total of 89 different peaks were identified, 48 of which were up-regulated and 41 were down-regulated (Table S7). However, 86 peaks were located in the intergenic region and only 3 peaks were located in the promoter region (Figure S2). One of the gene modules (Smechr0702191) was annotated as histone H2A and the others were annotated as uncharacterized proteins (Table 3).

## 3. Discussion

In the present study, our results showed that SmTCP7a, a transcriptional factor, positively regulated bacterial wilt resistance. Furthermore, ChIP-seq results identified several potential genes that were regulated by SmTCP7a during the eggplant response to bacterial wilt.

TCP factors regulate plant resistance through various ways, such as targeting pathogenic effectors, interconnecting hormonal networks, utilization of the circadian clock and transcriptional control [9]. To better understand the defense response of plants, it is important to identify which TCP family members regulate downstream target genes during pathogen infection. Several TCP target genes are involved in plant development, plant hormone biosynthesis, cold tolerance, disease and insect defense [31].To the best of our best knowledge, this study is the first to report genome-wide identification of binding sites for TCP transcription factors during plant disease resistance by ChIP-seq. Our results showed that SmTCP7a positively regulated bacterial wilt resistance in eggplant. In our previous report, SmEDS1 also positively regulated bacterial wilt resistance in eggplant, and SmEDS1 can directly interact with SmTCP7a [26,27]. To discover SmTCP7a target genes in relation to bacterial wilt, highly enriched peaks were detected by ChIP-seq. A total of 4378 and 6555 peaks were identified in R0 h and R48 h samples, respectively, but only 2.62% and 1.59% of these peaks were located in promoter regions with 92 and 91 gene modules, respectively. The percentage of peaks in the promoter region was lower than other results [2,15,30] and this difference may be due to non-specific binding by the polyclonal antibody against SmTCP7a.

DNA binding motifs within the peaks in promoter regions were detected by Homer. The motifs between R0 h and R48 h were different. This result suggests that after inoculation with *R.* *solanacearum*, SmTCP7a regulated different genes to produce wilt resistance in eggplant. Based on the TCP domain difference, TCP families can be divided into class I and class II. Although both TCP classes can recognize some common cis-elements [32], the conserved DNA binding sites of class I and class II are quite distinct [1]. Several results have shown that class I TCPs, such as TCP8 [8,11], TC9 [10], TCP14 [20], TCP15 [26] and TCP23 [27], play a key role in plant disease resistance. Our polygenetic tree results showed that SmTCP7a is a class I TCP. Motif analysis also showed that the best match motif in R0 h was G(T/C)(G/T)CGGGCCCCC. In R48 h, two motifs (TCP23 and PCF5) were identified as TCP binding motifs in JASPAR CORE plants. TCP23 also belongs to class I [32]. This result indicates that although the polyclonal antibody against SmTCP7a exhibited non-specific binding, the detected peaks are credible. However, the interaction between SmTCP7a and the promoters of these genes should be verified by EMSA or yeast one-hybrid experiments. 

Several of the peak-related genes have been reported to be involved in the plant disease response, namely, anthranilate synthase alpha subunit 1 (Smechr0600250), histone H2A (Smechr0702191), TCP9-like (Smechr0802294) transcription factor and R1B-23 (Smechr1100895). Based on previous reports, the anthranilate synthase gene can be induced by wound and pathogenic bacterial infection, and the production of tryptophan is part of the Arabidopsis defense response [33]. The histone H2A variant H2A.Z has been shown to interact with SWR1 to control immunity in Arabidopsis [34,35]. In future studies, the expression level and function of histone H2A during the eggplant response to bacterial wilt will be studied. The KEGG analysis for R48 h also showed that several pathways, such as glutathione biosynthesis, phenylpropanoid biosynthesis, MAPK signaling pathway, plant hormone signal transduction and plant-pathogen interactions were involved in plant biotic resistance. This result indicates that after inoculation with *R.* *solanacearum*, SmTCP7a regulates defense-related genes to respond to and resist infection. 

## 4. Materials and Methods

### 4.1. Plant Materials and Growth Conditions

The resistant eggplant inbred line “E35-1” [25,27] was planted in a small pot (4.5 inches). The growing material comprised sterile vermiculite and clay mixed in a 3:1 volume/volume ratio. The seedlings were grown in a temperature of 28 °C/25 °C (day/night) with a 16 h light/8 h dark photoperiod. After 4-week culture, when the seedlings were at the four-leaf stage, the culture was incubated with the *R.solanacearum* P2 strain. The root of the eggplant was cut using a scalpel and then inoculated with 50 mL of *R. solanacearum* (1.0 × 108 cfu). Then the eggplant was grown in a temperature of 32 °C/30 °C (day/night). At 0 and 48 h post-inoculation (hpi), the roots of five eggplants were collected and washed with water, then immediately frozen in liquid nitrogen and stored at −80 °C. All treatments were repeated two times.

### 4.2. Bioinformation Analysis of SmTCP7a

SmTCP7a (Smechr0103973) MW, theoretical isoelectric point, protein hydrophobicity and the aliphatic amino acid index were determined using the PortParam online tool available from ExPASy (https://expasy.org/proteomics (20 June 2022). Unrooted neighbor-joining phylogenetic trees were constructed using MEGA software with 1000 bootstrap replicates.

### 4.3. Expression Analysis and VIGS

The expression level of SmTCP7a was analyzed to determine the function of SmTCP7a during the eggplant response to *R. solanacearum*. After inoculation with *R.* *solanacearum*, SmTCP7a expression level was detected at the root of R and S eggplant. After the pTRV2 vector was digested by *Sma* I, 150 bp SmTCP7a was cloned into the pTRV2 vector by in-fusion methods. The primers are listed in Table S8. 

The pTRV2-SmTCP7a, pTRV2, and pTRV1 vectors were then transferred into *Agrobacterium tumefaciens* strain GV3101. After the pTRV1 equivalent was mixed with pTRV2-SmTCP7a, the mixture was infiltrated into four leaves of seedlings using a 1 mL needleless syringe. After injection, the samples were treated in the dark for one day and then inoculated with *R. solanacearum*. Each treatment had at least 10 biological replicates. The plant was grown in a temperature of 32 °C/30 °C (day/night).

### 4.4. Subcellular Localization and Transactivation Analysis of SmTCP7a Protein

The SmTCP7a CDS without a termination codon was cloned into the pCAMBIA1301-35S-EYFP vector by in-fusion. The recombinant vector was transformed into the *A. tumefaciens* strain GV3101. Transient expression assays were conducted in *N. benthamiana* leaves. Transactivation by SmTCP7a was examined in a strain of *Saccharomyces cerevisiae.* The effector vector was pGreenII 62SK-GAL4BD and the reporter vector was pGreeII-0800-5*GAL4- TATA-Luc (Figure S3). The effector/reporter rotation was 1:9 and the transient expression assays were conducted in *N. benthamiana* leaves. Luciferase assays were performed with the Dual-Luciferase^®^ Reporter Assay System (Promega, E1960) using a TECAN spark microplate reader.

### 4.5. Production of a Polyclonal Antibody against SmTCP7a and Western Blot Analysis

The SmTCP7a CDS without a termination codon was cloned into the pET-28a(+) vector. After the SmTCP7a protein was purified, it was injected into a healthy New Zealand white rabbit. The polyclonal antibody against SmTCP7a was purified in accordance with the methods of Iqbal [2]. The purity of the antibody was then evaluated using SDS-PAGE and stored at −20 °C. Western blot analysis was carried out in accordance with the method of Li to examine the quality and specificity of the antibody for the ChIP-Seq assay [36].

### 4.6. ChIP-Seq Library Preparation and Data Analysis

Approximately 4g of eggplant root was cross sectioned using scissors and then cross linked with 1% formaldehyde in a vacuum for 15 min. After the samples were ground to powder in liquid nitrogen, the chromatin complexes were isolated following [2]. Later, the chromatin was sonicated to shear the DNA into 100–500 bp fragments using a Branson digital probe sonicator. Subsequently, the sonicated DNA was incubated with a polyclonal antibody developed against the SmTCP7a. The control treatment, which was treated with pre-immune sera, was used for each experiment. DNA-antibody complexes were then precipitated, and the DNA was recovered by dissociating the complexes. ChIP-seq library construction and high-throughput sequencing were carried out by using the Illumina HiSeq2000.

After the reads were filtered by fastqc, the clean reads were aligned to the eggplant reference genome HQ1315 [37] using bwa-mem2. The experiment was conducted with two conditions: the control library and the antibody-treated library. MACS with standard parameters was used for peak calling to locate the enriched regions of binding sites. All the binding peaks in the genomic locations were sorted into intergenic, intron, promoter and exon regions using the ChIPseeker R package [29]. A motif search was performed using Homer. The different peaks in R0 h and R48 h were analyzed by using the DiffBind R package with default parameters.

## 5. Conclusions

In this study, our results showed that the SmTCP7a transcriptional factor was inducible and up-regulated after *R. solanacearum* treatment. Furthermore, the VIGS result showed that SmTCP7a positively regulated bacterial wilt resistance. The potential target genes were analyzed by ChIP-seq. The identification of DNA motifs and regulatory genes provides a new approach for understanding the molecular mechanism of SmTCP7a during the eggplant response to *R. solanacearum*.

## Figures and Tables

**Table 1:** Summary of ChIP-seq reads from eight different libraries.

Treatment	Condition	Clean Reads	Genome Mapped Reads
R0 h-1	Control	37245858	34352758
	Antibody treated	36252688	27346750
R0 h-2	Control	45348560	40775010
	Antibody treated	45783576	27961628
R48 h-1	Control	39735838	30495054
	Antibody treated	44687510	37500882
R48 h-2	Control	47621298	37103632
	Antibody treated	49651010	43919431

**Table 2:** ChIP-seq data reveals peak-associated genes regulated by SmTCP7a.

Gene	NR Annotation	Sample
Smechr0702191	Histone H2A	R0 h
Smechr0601128	protein argonaute 4	
Smechr0501578	WRKY transcription factor protein 1	
Smechr0500083	ubiquitin-conjugating enzyme E2	
Smechr0400288	glutathione S-transferase parC	R48 h
Smechr0702191	Histone H2A	
Smechr0800428	subtilisin-like protease SBT3.3	
Smechr0802294	transcription factor TCP9-like	
Smechr1100895	putative late blight resistance protein homolog R1B-23	

**Table 3:** The difference in peak-associated genes between R0 h and R48 h.

Gene ID	Annotation	Regulation
Smechr0300595	uncharacterized protein LOC109121249	Up
Smechr0103296	uncharacterized protein LOC107855847 isoform X1	Up
Smechr0702191	Histone H2A	down

## Supplementary Materials

The following supporting information can be downloaded at: https://www.mdpi.com/article/10.3390/ijms23126844/s1.
