# Supplementary material for: Genome-Wide Identification of Binding Sites for SmTCP7a Transcription Factors of Eggplant during Bacterial Wilt Resistance by ChIP-Seq
Source: Int J Mol Sci. 2022 Jun 20;23(12):6844. doi: 10.3390/ijms23126844 (PMC9224693; doi:10.3390/ijms23126844)
Supplement: Supplementary file 1 [file ijms-23-06844-s001.zip › Table S8.pdf]

Table S8 The primes used in the present study.

Primer	F 5’-3’	R 5’-3’
qSmTCP7a	GCGAAACTCCAAACTTgCCCATA	GGAAAGCGTTTACGGTCTGAGGA
pTRV-2S	CGCGTCTCGAGGCCCGGGAT	CTTCGGGACATGCCCGGGTAT
mTCP7a	GGCGACGTCGGTAGAAC	ACGTCTTCCTCTTCCATCA
