# Supplementary figures and images for: Genome-Wide Identification of Binding Sites for SmTCP7a Transcription Factors of Eggplant during Bacterial Wilt Resistance by ChIP-Seq
Source: Int J Mol Sci. 2022 Jun 20;23(12):6844. doi: 10.3390/ijms23126844 (PMC9224693; doi:10.3390/ijms23126844)

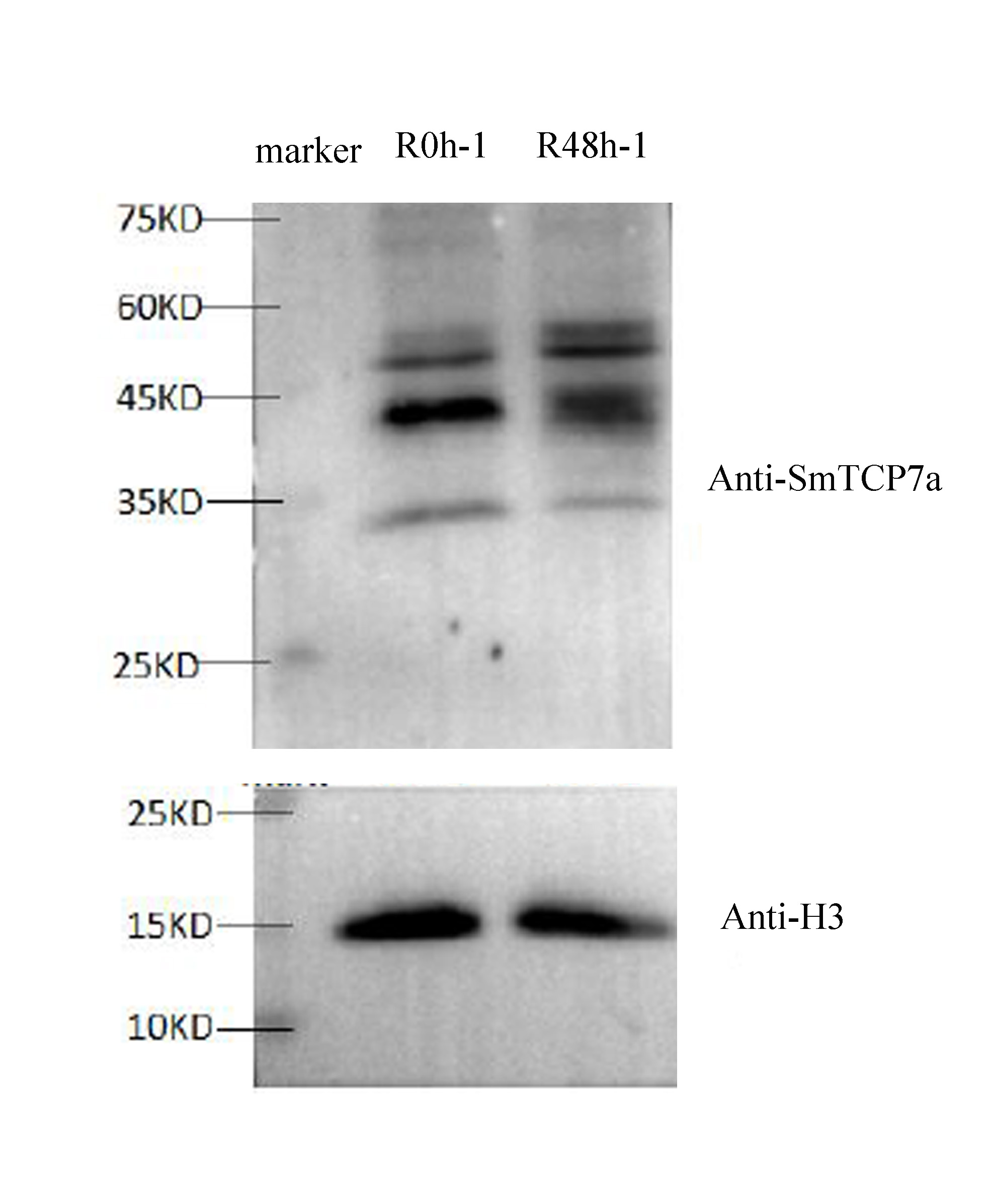

Supplement: Supplementary file 1 [file ijms-23-06844-s001.zip › Figure S1.jpg]

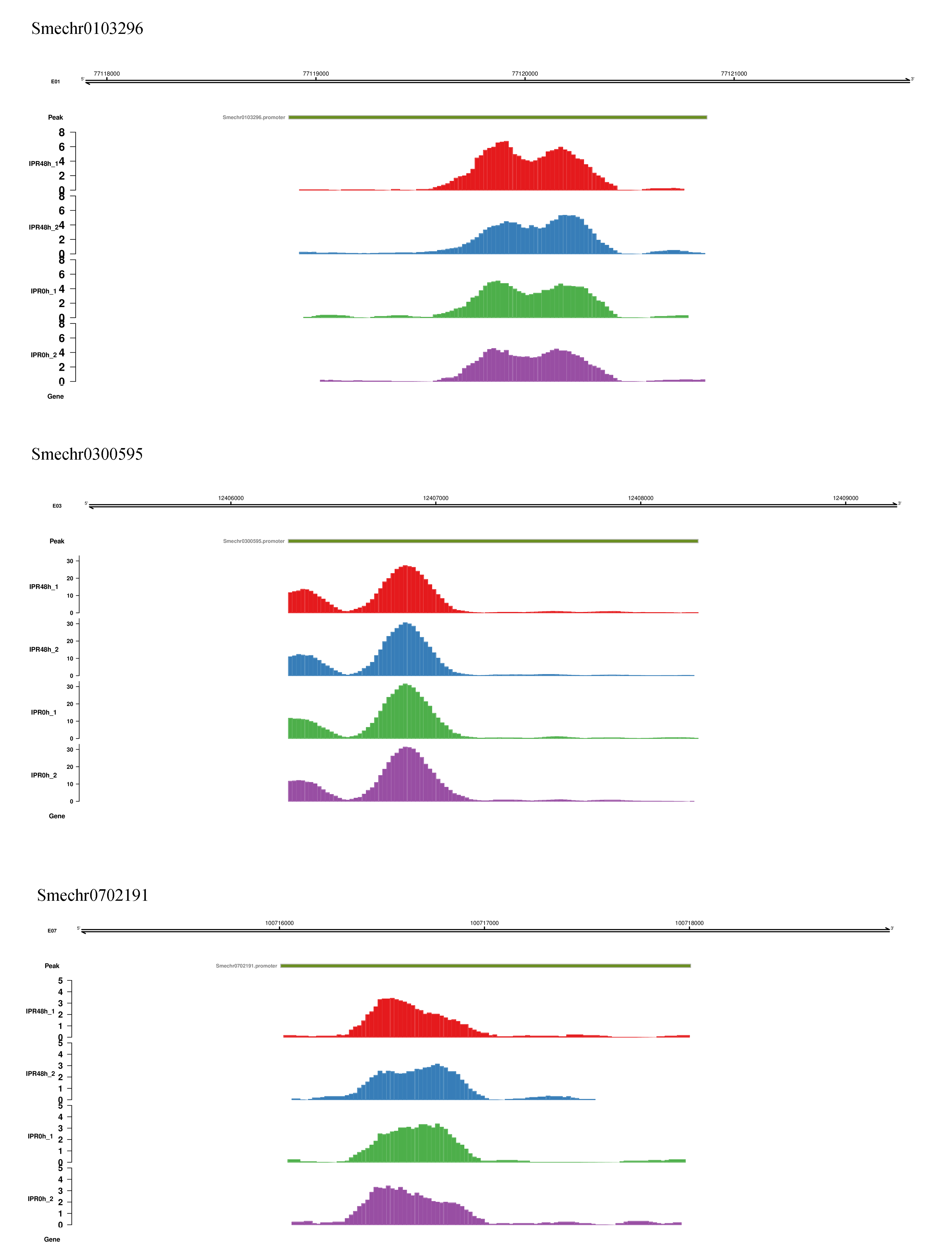

Supplement: Supplementary file 1 [file ijms-23-06844-s001.zip › Figure S2.jpg]

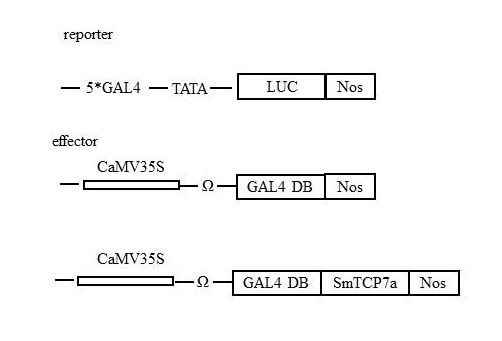

Supplement: Supplementary file 1 [file ijms-23-06844-s001.zip › Figure S3.jpg]
